# Perivascular epitheloid cell tumor (PEComa) mimicking retroperitoneal liposarcoma

**DOI:** 10.1186/1477-7819-12-3

**Published:** 2014-01-06

**Authors:** Moritz Wildgruber, Karen Becker, Marcus Feith, Jochen Gaa

**Affiliations:** 1 Department of Diagnostic and Interventional Radiology, Klinikum Rechts der Isar, TU München, Ismaningerstrasse 22, München D-81675, Germany; 2 Department of Pathology, Klinikum Rechts der Isar, Technische Universität München, Munich, Germany; 3 Department of Surgery, Klinikum Rechts der Isar, Technische Universität München, Munich, Germany

**Keywords:** PEComa, Mesenchymal tumor, Liposarcoma, mTOR signaling

## Abstract

PEComas are a collection of generally rare tumors, defined by the World Health Organization as ‘mesenchymal tumors composed of histologically and immunohistochemically distinctive perivascular epitheloid cells’. We describe the case of retroperitoneal PEComa with a liposarcoma-like appearance on cross-sectional imaging, but distinctive immunohistochemistry revealing the correct diagnosis.

## Background

Perivascular Epitheloid Cell tumors (PEComas) are rare mesenchymal tumors that can appear at various sites in the body and can be associated with the tuberous sclerosis complex. They can possess a heterogeneous appearance, ranging from poorly differentiated soft-tissue tumors to sclerosing masses. Lipid-rich PEComas are uncommon. We present the case of a large lipid-rich PEComa primarily misdiagnosed as a liposarcoma on computed tomography and magnetic resonance imaging. Histology, however, revealed a lipid-rich PEComa without any signs of malignancy, which required no further treatment following surgical resection.

## Case presentation

**Figure 1:**
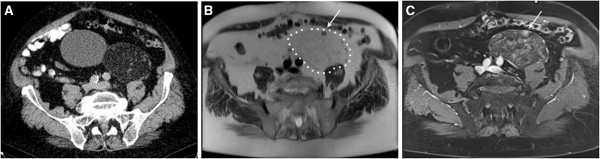
Computed tomography (A) and magnetic resonance imaging findings (B and C) demonstrate an ovoid encapsulated retroperitoneal mass, which contained predominantly fat and a few solid hypervascularized nodules (arrows).

A 75-year-old man was referred for abdominal computed tomography (CT) due to a poorly defined liver mass, detected by the primary care physician on ultrasound screening, which turned out to be a hemangioma. As an accidental finding, the contrast-enhanced CT scan additionally showed an 8 × 11 × 15 cm large retroperitoneal mass in the lower left abdomen adjacent to the sigmoid colon and the left psoas muscle (Figure 1A). The density measurements revealed that the thinly encapsulated mass consisted predominantly of fat with a few solid nodular structures. Contrast-enhanced magnetic resonance imaging (MRI) confirmed the lesion to be composed predominantly of fat with a few solid hypervascularized nodules (Figure 1B and C). The mass was palpable through the abdominal wall but painless. Laboratory findings indicated a mild anemia (hemoglobin 12.9 g/dl) but were otherwise unremarkable. The patient had no history of malignant or infectious disease.

**Figure 2:**
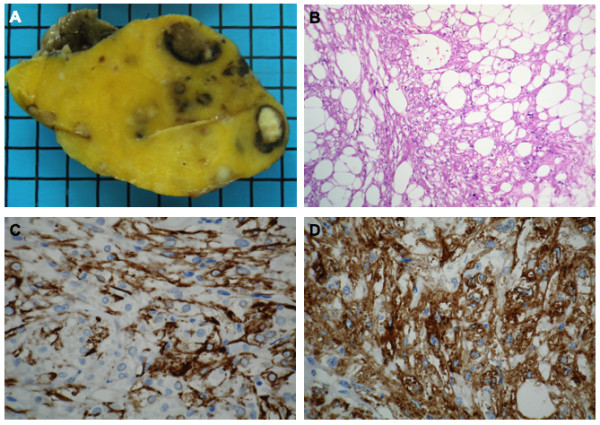
Macroscopic appearance (A) and histology with H&E staining (B) and immunohistochemistry (IHC) for smooth muscle actin (C) and human melanoma black (HMB)-45 (D).

Due to the imaging findings, a retroperitoneal liposarcoma was suspected and the patient underwent surgery with resection of the mass and the adjacent sigmoid colon. Intraoperatively, the tumor showed firm adhesions both with the mesentery of the sigmoid colon as well as with the adjacent small bowel mesentery. During adhesiolysis, several tumor feeding vessel originating from the inferior mesenteric artery were identified and clamped. Due to the close proximity of the tumor to the sigmoid colon, the latter was resected *en bloc* together with the mass and a side-to-side descendo-rectostomy was performed. Macroscopic appearance as well as histology and immunohistochemistry of the mass are shown in Figure 2.

Macroscopic evaluation of the surgical specimen confirmed the predominance of fat within the thinly encapsulated tumor (Figure 2A). Histology demonstrated the presence of large-sized lipid vacuoles and small, disseminated islands of lipoblasts (Figure 2B). Variable-sized nests of epitheloid cells were spread over the tumor. Immunohistochemically, these cells showed only weak staining for melan-A, desmin and smooth muscle actin (SMA) (Figure 2C). S-100 staining was negative but strong reaction was found for human melanoma black HMB-45 (Figure 2D). MiB1 staining revealed a proliferation rate < 1% and no mitoses were observed. A fluorescence *in situ* hybridization (FISH) for MDM2 gene amplification was negative, thus a liposarcoma was ruled out and the final diagnosis was lipid-rich perivascular epitheloid cell tumor (PEComa) without any signs of malignancy. The patient recovered well from surgery and did not require any further adjuvant treatment.

## Discussion

PEComas are a rare mesenchymal tumor entity incorporating angiomyolipomas, clear ‘sugar’ cell tumors, lymphangioleiomyomas and histologically and immunophenotypically similar tumors occurring at various soft-tissue and visceral sites, and can be associated with the tuberous sclerosis complex [1,2]. Distinctive epitheloid tumor cells show a focal association with blood vessels and express melanocytic and smooth muscle markers, the most sensitive being HMB-45 [3,4].

PEComas have been described at various extrarenal locations including the mesentery, urinary bladder, liver, pancreas, rectum, heart and lung as well as in the bone [5-11]. Retroperitoneal localization of PEComas has been described [12,13], but a liposarcoma-like aspect of the tumor in this localization as in our case is extremely rare. PEComas occurring in these non-classical locations have been termed ‘perivascular epitheloid cell tumors not otherwise specified’ (PEComa-NOS) [14,15].

The imaging signature of PEComas is highly variable, although a few common features have been described. Mostly, PEComas have well defined borders and are of regular shape. The enhancement on cross-sectional imaging after contrast agent administration is usually inhomogeneous due to the heterogeneous composition of the tumor [16].

The degree of malignancy is highly variable in PEComas, but as with other sarcomas, the size of the primary tumor as well as the mitotic rate seem to be the most reliable prognostic factors associated with recurrence after surgical resection. Bleeker *et al*. have recently established a suggestion for risk stratification and treatment strategies [14]. If possible, surgical resection is the treatment of choice for PEComas and, as activation of the mTOR signaling pathway is common in these tumors, mTOR inhibitors such as rapamycin have been successfully applied as medical treatment . Although no large series of patients treated with mTOR inhibitors exist, preliminary data suggest that complete response is possible [17].

With a size > 5 cm but absent further risk factors such as infiltrative growth pattern, high nuclear grade and cellularity, mitotic rate > 1/50 HPF, necrosis or vascular invasion, the tumor in our patient was stratified as ‘uncertain malignant potential’ according to the modified Folpe criteria [14]. According to these criteria, adjuvant therapy may be of benefit in patients with high risk of recurrence, which was not considered to be present in our patient. However, regardless of the postoperative strategy employed, long-term surveillance should be at its core, as recurrences have been reported more than five years following resection [14]. Our patient is scheduled for clinical follow-up evaluation every six months for the first five years and, similar to sarcoma patients, with contrast-enhanced MRI every six months in the first three years and subsequent annual MRI examinations thereafter. Today, one and a half years after surgical resection, the patient is free of recurrence.

## Conclusion

Although a rare tumor entity, PEComas are occasionally found as usually well-circumscribed tumors, often located in the abdomen, retroperitoneum or the pelvis. Besides surgical resection as the treatment option of choice, clinicians should be aware of new treatment strategies using inhibitors of the mTOR signaling pathway, which is commonly activated in PEComas.

## Consent

Written informed consent was obtained from the patient for publication of this case report and any accompanying images. A copy of the written consent is available for review by the Editor-in-Chief of this journal.

## Abbreviations

CT: Computed tomography; HPF: High-power field; MRI: Magnetic resonance imaging; mTOR: Mamallian target of rapamycin; PEComa: Perivascular epitheloid cell tumor.

## Competing interest

The authors declare that they have no competing interests.

## Authors’ contributions

All authors we involved in the clinical care of the patient described in this case and contributed similarly to preparation and review of the manuscript. All authors read and approved the final manuscript.
